# The Antioxidant, Antibacterial and Cell-Protective Properties of Bioactive Compounds Extracted from Rowanberry (*Sorbus aucuparia* L.) Fruits In Vitro

**DOI:** 10.3390/plants13040538

**Published:** 2024-02-16

**Authors:** Mara Aurori, Mihaela Niculae, Daniela Hanganu, Emoke Pall, Mihai Cenariu, Dan Cristian Vodnar, Nicodim Fiţ, Sanda Andrei

**Affiliations:** 1 Department of Preclinical Sciences, Faculty of Veterinary Medicine, University of Agricultural Sciences and Veterinary Medicine Cluj-Napoca, 400372 Cluj-Napoca, Romania; mara.aurori@usamvcluj.ro; 2 Department of Clinical Sciences, Faculty of Veterinary Medicine, University of Agricultural Sciences and Veterinary Medicine Cluj-Napoca, 400372 Cluj-Napoca, Romania; mihaela.niculae@usamvcluj.ro (M.N.); emoke.pall@usamvcluj.ro (E.P.); mihai.cenariu@usamvcluj.ro (M.C.); 3 Department of Pharmacognosy, Faculty of Pharmacy, University of Medicine and Pharmacy “Iuliu Haţieganu”, 400372 Cluj-Napoca, Romania; dhanganu@umfcluj.ro; 4 Department of Food Science, Faculty of Food Science and Technology, University of Agricultural Sciences and Veterinary Medicine Cluj-Napoca, 400372 Cluj-Napoca, Romania; dan.vodnar@usamvcluj.ro; 5 Department of Paraclinical Sciences, Faculty of Veterinary Medicine, University of Agricultural Sciences and Veterinary Medicine Cluj-Napoca, 400372 Cluj-Napoca, Romania; nfit@usamvcluj.ro

**Keywords:** *Sorbus aucuparia* L., rowanberry, fruit extract, phenolic compounds, carotenoids, antioxidant, antibacterial, cytoprotection, renal cell injury, in vitro

## Abstract

Considering that *Sorbus aucuparia* fruits have been underutilized despite their tremendous potential, this study aimed to correlate the in vitro antioxidant, antibacterial and cell-protective abilities of fruit extracts derived from *Sorbus aucuparia* Romanian cultivars with their phytochemical composition. Therefore, following the preparation of ethanolic and carotenoid extracts, phytochemical screening was performed using UV–Vis and HPLC-DAD-ESI-MS methods. The antioxidant activity was analyzed using DPPH and FRAP tests. As the results revealed high contents of bioactive compounds (polyphenols 1.11 mg GAE/g DM, flavonoids 430.06 µg QE/g DM and carotenoids 95.68 µg/g DM) and an important antiradical action (DPPH 24.51 mg/mL and FRAP 0.016 µM TE/mL), we chose to further examine the fruits’ biological properties. The antibacterial capacity was assessed employing agar well diffusion and broth microdilution techniques, with fruits displaying an intense activity against MSSA, MRSA and *Enterococcus faecalis*, but also *E. coli* and *Pseudomonas aeruginosa*. The cell-protective activity was analyzed on gentamicin-stressed renal cells, through MTT and Annexin V-FITC assays. Importantly, a significant increase in viability was registered on stressed cells following extract administration in low doses; nevertheless, viability was noticed to decline when exposed to elevated concentrations, potentially due to the cumulative actions of the extract and gentamicin. These findings offer novel light on the antibacterial activity of *Sorbus aucuparia* Romanian cultivars, as well as their cell-protective ability in renal cell injury.

## 1. Introduction

The public’s rising comprehension of nutritious foods that enhance health creates new obstacles and scientific challenges. Fruits are regarded as healthful foods, owing to their high content of important nutrients [1]. Wild berries, specifically blueberries, raspberries, blackberries, cranberries, forest strawberries and cherries, are valuable because, in addition to containing an abundance of bioactive compounds that can prevent numerous illnesses, they possess unique flavors and are thus easily consumed raw or as refined food products [2]. Nevertheless, despite their high phytonutrient content, a multitude of woodland berries remain neglected mainly due to their disagreeable savor sensed by consumers. Therefore, researching and integrating these unexplored fruits into both cultivation and consumption strategies could result in considerable improvements in human wellness, nutrition, and sustainability in nature [3].

*Sorbus aucuparia* L. represents one such under-exploited forest fruit, commonly recognized as rowanberry or mountain ash, being part of the *Rosaceae* family. *Sorbus aucuparia* L. is a slender tree that can reach a height of 15–20 m and thrives in the Northern Hemisphere at varying altitudes, spreading from the European Atlantic coastlines to the Kamchatka Peninsula and the eastern part of China [4,5,6]. Its fruits have a spherical shape and are frequently reddish orange, containing 2–3 elongated stones [7,8]. Throughout history, *Sorbus aucuparia* L. berries have been utilized in the treatment of various ailments, including gastrointestinal, pulmonary, renal, hepatic and cardiovascular problems. The use of these berries as diuretics, anti-inflammatory, antidiabetic, laxative, antibacterial, antipyretic and vasodilating agents has also been documented [9,10,11]. These fruits were also incorporated into food supplements for being used on a daily basis; however, due to their characteristic bitter and astringent flavor, they were generally overlooked or used sporadically [12].

Previous studies have shown that *Sorbus aucuparia* L. fruits contain a substantial number of bioactive constituents, including polyphenols, carotenoids, organic acids, vitamins, microelements and phytoalexins [13,14]. The most prevalent active chemicals are represented by polyphenols, from which proanthocyanidins and chlorogenic and neochlorogenic acids dominate the composition of *Sorbus aucuparia* L. berries [5,9,15,16]. Additionally, prior studies revealed that the carotenoid content in *Sorbus aucuparia* L. is comparable to that discovered in carrots [17,18]. Furthermore, these berries hold an increased quantity of vitamin B2, E and C, the latter being found in a similar manner to that of strawberries [18]. Although current studies show that these berries are enriched with bioactive substances, more research is necessary to fully characterize their phytochemical profile [9].

Prior research on the biological activities of *Sorbus aucuparia* L. has been quite limited. To the best of our knowledge, studies have been focused on its antiproliferative, antibacterial [9,11,19] and digestive effects [20,21], with a stronger emphasis on its cardiovascular and antidiabetic effects [15,22,23,24]. Additionally, the antioxidant activity of *Sorbus aucuparia* L. has also been discovered as an essential element of its health-enhancing properties [9,10,17].

Kidneys are small organs which regulate various processes that contribute to homeostasis. One of their main functions represents the removal of xenobiotics—potentially nephrotoxic substances—that are transported to the organ in relatively high quantities, rendering them a prime candidate for drug toxicity. Due to their high metabolic activity, the proximal tubules are the most susceptible to the nephrotoxic effects of xenobiotics and pharmaceuticals [25,26]. Oxidative stress represents a key cause of drug-induced nephrotoxicity, as evidenced by increased generation of reactive oxygen species and mitochondrial dysfunction. Renal tubular cells are especially vulnerable to oxidative stress, eventually causing apoptosis and necrosis [27]. Given this concern, reducing the toxicity of medications that exacerbate oxidative stress could be accomplished by exploiting natural riches such as therapeutic plants, which constitute a vast supply of natural antioxidants.

Since *Sorbus aucuparia* L. fruits have proven to be a valuable reservoir of bioactive elements and have been poorly utilized despite having great potential, we assumed it was vital to further characterize the profile of their natural compounds in correlation to their implications at the cell level. Specifically, we focused on analyzing the carotenoid fraction of these fruits, which are antioxidant lipophilic compounds that have not been previously explored for their advantageous effects in renal disease. Consequently, the present paper aims to discover and measure the bioactive constituents of *Sorbus aucuparia* L. Romanian fruits along with evaluating the in vitro antioxidant, antibacterial and nephroprotective impacts on gentamicin-stressed primary mouse kidney cells, as the last property may aid in the discovery of new remedies for minimizing drug-induced nephrotoxicity.

## 2. Results

### 2.1. Quantitative Determination of Total Polyphenols, Flavonoids and Carotenoids of Sorbus aucuparia L. Fruit Extracts

The study’s first goal was to quantify the major bioactive compounds present in *Sorbus aucuparia* L. cultivars from Cluj County, Romania, namely polyphenols, flavonoids and carotenoids.

As a result of the ethanolic extract analysis, the total phenolic content was determined to be 1.39 ± 0.046 mg GAE/mL and the total flavonoid concentration was 537.58 ± 3.255 µg QE/mL. In relation to the fruit’s weight, the total content of phenolic compounds was 1.11 ± 0.030 mg GAE/g DM and the total flavonoids were 430.06 ± 2.603 µg QE/g DM. The evaluation of the specific carotenoid extraction yielded a value of 95.68 ± 0.297 µg/g DM for total carotenoid concentration.

### 2.2. HPLC-DAD-ESI-MS Characterization of Phenolic Compounds of Sorbus aucuparia L. Fruit Extract

The identification of phenolic compounds using HPLC-DAD-ESI-MS analysis was performed by contrasting the UV–Vis spectrum, the retention period and the level of each particular peak to standards and previously released data. Consequently, ten compounds from three distinct subclasses were discovered. The subclass of phenolic acids was the most prominent, consisting of gallic acid glucoside, chlorogenic acid, neochlorogenic acid, cryptochlorogenic acid and ferulic acid. The flavonol subclass was composed of Q 3,4′-O-diglucoside, Q 3-O-glucoside and Q 3-O-rutinoside, whereas Cy 3-O-(caffeoyl-glucoside) and Cy 3-O-glucoside were the representatives of the least abundant subclass, that of anthocyanins.

Of all detected compounds, the greatest value of GAE was identified in chlorogenic acid (704.792 µg/mL), followed by neochlorogenic acid (376.610 µg/mL), with the lowest levels being reported in Q 3-O-rutinoside and Cy 3-O-(caffeoyl-glucoside) (12.198 and 3.212 µg/mL, respectively). Furthermore, the extract’s overall phenolic concentration was 1.40 mg GAE/mL. This result is consistent with that obtained using the Folin–Ciocalteu method (1.40 mg GAE/mL vs. 1.39 mg GAE/mL).

**Figure 1:**
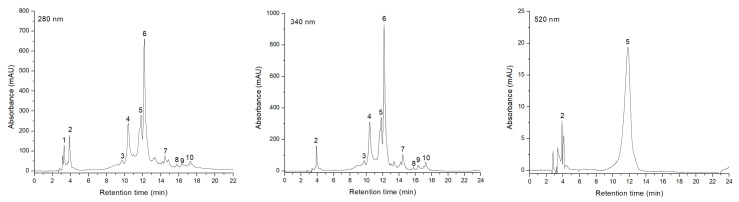
HPLC Chromatography of phenolic compounds extracted from *Sorbus aucuparia* L. fruits; detection and measurement of phenolic acids at λ = 280 nm, flavonoids at λ = 340 nm and anthocyanins at λ = 520 nm; the peak values correspond to the chemicals present in Table 2: 1-Gallic acid-glucoside, 2-Cy 3-O-(caffeoyl-glucoside), 3-Cryptochlorogenic acid, 4-Neochlorogenic acid, 5-Cy 3-O-glucoside, 6-Chlorogenic acid, 7-Q 3,4′-O-diglucoside, 8-Rutin, 9-Q 3-O-glucoside, 10-Ferulic acid.

These outcomes can be followed in Figure 1 and Table 1.

### 2.3. The Antioxidant Activity of Sorbus aucuparia L. Fruit Extract

An ultimate IC_50_ value of 24.51 ± 0.577 mg/mL of extract was found by measuring the antioxidant capacity using the Radical Scavenging Activity (DPPH) assay. Concurrently, using the Ferric Reducing Antioxidant Power (FRAP) technique, an outcome of 0.016 ± 1.047 was provided, being revealed in µM TE/mL of extract.

### 2.4. The Antibacterial Capacity of Sorbus aucuparia L. Fruit Extract

#### 2.4.1. Antibacterial Activity by Agar-Well Diffusion Method

The results obtained for the in vitro antimicrobial potential of *Sorbus aucuparia* L. extract are displayed in Table 2 (diameters of inhibition zone) and Table 3 (MIC index calculated based on MBC and MIC values).

*Sorbus aucuparia* L. extract showed an important antimicrobial activity towards both Gram-positive and Gram-negative bacteria included for testing (Table 2). Of particular interest, the in vitro efficacy against MSSA—Methicillin-Susceptible *Staphylococcus aureus*, MRSA—Methicillin-Resistant *Staphylococcus aureus*, *Escherichia coli* and *Enterococcus faecalis* was found to be significantly higher (*p* < 0.05) compared to the positive controls (gentamicin in case of Gram-negative, and amoxicillin-clavulanic acid for the Gram-positive). Furthermore, the extract inhibitory activity on *Pseudomonas aeruginosa* reference strain growth was similar to gentamicin (*p* > 0.05). The least susceptible was the strain of *Bacillus cereus* with the diameter of the inhibition zone significantly lower (*p* < 0.05) compared to gentamicin. These results are below those previously published that present Gram-negative bacteria’s lower sensitivity associated with their bacterial wall particularities [5].

#### 2.4.2. Antibacterial Activity by Broth Microdilution Method

For each tested bacterial strain, MIC and MBC values were determined using the broth microdilution method and the results are presented in Table 3. These data reflect *Sorbus aucuparia* L.’s superior efficacy against the Gram-positive bacteria, *S. aureus*, MRSA, *Bacillus cereus* and *Enterococcus faecalis* as the lowest tested concentrations (0.010 µg/uL) were able to inhibit and kill bacteria, respectively. Concentrations of 0.087 µg/uL and 0.043 µg/uL were required to exhibit antimicrobial efficacy against the Gram-negative strains *E. coli* and *Pseudomonas aeruginosa*. In the case of *Ps. aeruginosa*, the recorded results present the highest tested concentration (0.087 µg/uL). Still, this aspect is expected given the elevated intrinsic antimicrobial resistance described for this bacterium, while the determined value of the MIC index is encouraging. This value indicates that the activity is bactericidal (MBC/MIC ≤ 4). Furthermore, the same value 1 was established for all the tested bacteria, both Gram-positive and Gram-negative.

### 2.5. The Effect of Sorbus aucuparia L. Fruit Extract on Gentamicin-Stressed Primary Mice Renal Cells In Vitro

#### 2.5.1. Cell Viability Analysis by MTT Assay

Because fruits were demonstrated to contain high contents of bioactive compounds and showed a significant antioxidant activity, we tested whether they exert protective effects on mice renal epithelial cells in the context of gentamicin exposure. As such, the cultivated cells were separated into four distinct categories:

*Control (Ctrl) group*, which included non-treated cells;
*Gentamicin (GEN) group*, which was given antibiotic therapy in 3 separate doses, namely GEN1 = 100 μg/μL, GEN2 = 150 μg/μL and GEN3 = 200 μg/μL;
*Sorbus aucuparia (SA) group*, which received herbal therapy in 3 different concentrations, particularly SA1 = 4.8 μg/μL, SA2 = 10.7 μg/μL and SA3 = 19.1 μg/μL;
*Gentamicin* + *Sorbus aucuparia (GSA) group*, which received treatment with a mixture of both, namely GSA1 = 100 μg/μL gentamicin + 4.8 μg/μL *Sorbus aucuparia* L. extract, GSA2 = 100 μg/μL gentamicin + 10.7 μg/μL *Sorbus aucuparia* L. extract and GSA3 = 100 μg/μL gentamicin + 19.1 μg/μL *Sorbus aucuparia* L. extract.

**Figure 2:**
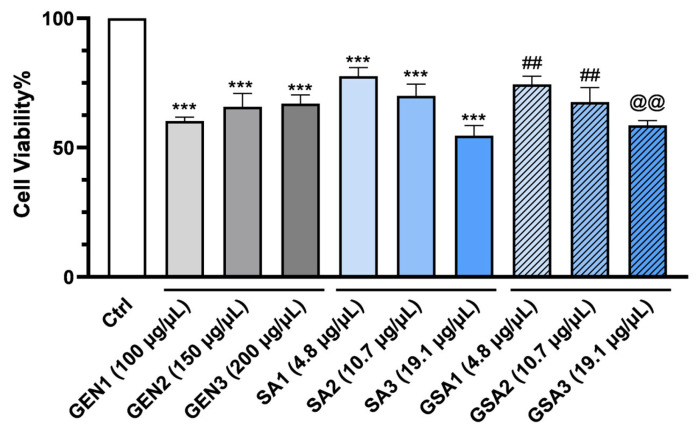
Results of MTT assay. *** *p* < 0.001 (vs. control)—cytotoxic activity of Gentamicin and *Sorbus aucuparia* L. extract in three different concentrations (GEN1-GEN3 and SA1-SA3) on primary mice renal epithelial cells; ^##^ *p* < 0.01 (vs. GEN1)—cell-protective effect of *Sorbus aucuparia* L. extract (GSA1 and GSA2) on gentamicin-stressed primary mice renal epithelial cells; ^@@^ *p* < 0.01 (vs. SA3)—cytotoxic activity of *Sorbus aucuparia* L. extract in high doses on primary mice renal epithelial cells. One-way ANOVA was employed for data analysis. The findings reflect the mean ± SD of three measurements.

After dividing the cells, the renal cell-protective activity was first evaluated by the MTT assay. As shown in Figure 2, all doses of *Sorbus aucuparia* L. extract determined a significant decrease in cell viability in a dose-dependent manner compared to the untreated group (*p* < 0.001). Although unexpected, the lowest dose of extract registered the highest percentage of viability (77.63 ± 0.36%). Moreover, all gentamicin concentrations determined a drastic decline in cell viability when compared to control (*p* < 0.001). The first concentration, which showed the least percentage of viability (60.3 ± 0.45%), was discovered to be the most injurious to the renal cells. Consequently, this concentration was selected for further analysis and interpretation. Importantly, it could be observed that the cells exposed to gentamicin and fruit extract together recorded a substantial higher percentage of viability when compared to gentamicin-only treated cells (*p* < 0.01), with the extract demonstrating a renal cell-protective impact at the first two concentrations (4.8 μg/μL and 10.7 μg/μL). Even more, the viability of these cells was comparable to that of extract-only treated cells (*p* > 0.05). Thus, we can assume that the low dosages of extract protect kidney cells against gentamicin stress. Unfortunately, there was no statistical difference in cell viability between the 19.1 μg/μL extract + gentamicin group and cells that received gentamicin treatment only (*p* > 0.05). This finding could be explained by the probability that in high doses, the extract exhibits pro-oxidant effects similar to gentamicin.

#### 2.5.2. Cell Apoptosis Measurement by Annexin V-FITC Staining

**Figure 3:**
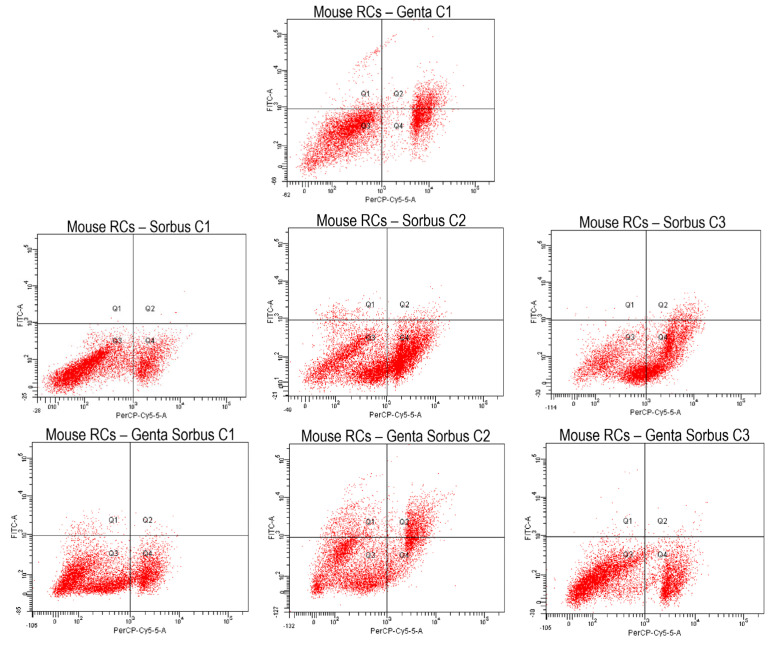
Cellular apoptosis by flow cytometry of Gentamicin (GEN 100 µg/µL), *Sorbus aucuparia* L. fruit extract (SA1-SA3) and Gentamicin + *Sorbus aucuparia* L. fruit extract (GSA1-GSA3) on mice kidney cells; Q1 = early apoptotic cells, Q2 = late apoptotic cells, Q3 = viable cells and Q4 = necrotic cells.

**Figure 4:**
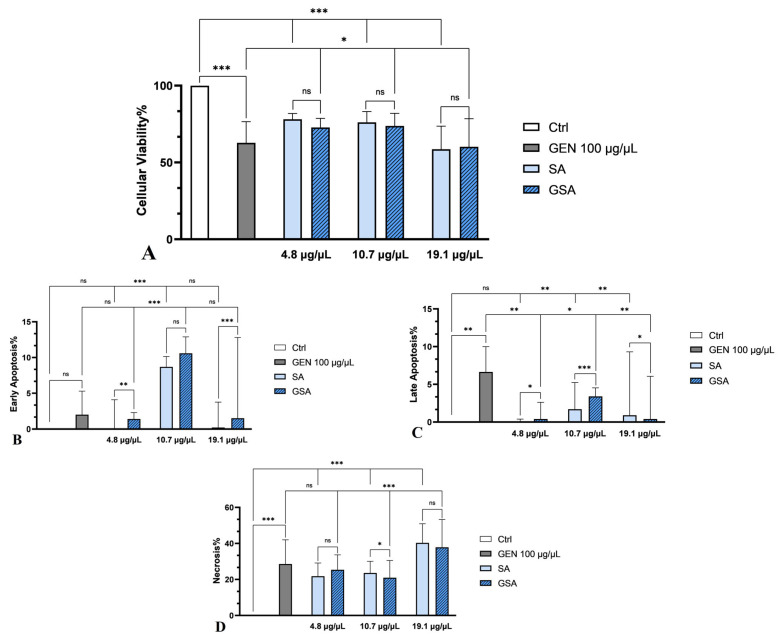
Flow cytometry analysis of cell apoptotic rate. (**A**) Viable cells, (**B**) Early apoptotic cells, (**C**) Late apoptotic cells, (**D**) Necrotic cells; * *p* < 0.05, ** *p* < 0.01, *** *p* < 0.001, ns = non-significant. The data were analyzed using one-way ANOVA. The outcomes indicate the mean ± SD of triplicate assessments.

Cell categories employed in the MTT experiment, namely Ctrl, GEN (100 µg/µL), SA1-SA3 and GSA1-GSA3, were subjected to apoptotic measures. Flow cytometry findings were obtained after PI and Annexin V affinity testing and fluorochrome labeling, as revealed in Figure 3 and Figure 4. Similar to MTT results, all *Sorbus aucuparia* L. extract doses determined a reduction in cell viability in comparison to control group (*p* < 0.001). As well, gentamicin group experienced an even more severe decline of viability when compared to untreated cells (*p* < 0.001). Following extract treatment to antibiotic-stressed cells, a notable rise of viable cells was seen at the first two dosages (4.8 μg/μL and 10.7 μg/μL) when compared to antibiotic-only treated cells (*p* < 0.05), determining a renal cell-protective impact as stated in MTT assay. Additionally, the number of viable cells was similar to that registered in the extract-only treated group (*p* > 0.05), reinforcing the hypothesis that the low dosages of extract possess renal cell-protective effects. Unpleasantly, the last dosage exhibited a cytotoxic activity, as the percentage of viable cells was significantly reduced even when compared to antibiotic-only treated group (*p* < 0.05). This may be due to the combined effects of gentamicin and carotenoids, which might operate as pro-oxidants in large quantities. Figure 4A illustrates these results graphically.

Furthermore, *Sorbus aucuparia* L. extract treatment in dose of 4.8 μg/μL did not determine a rise in early and late apoptosis when compared to control (*p* > 0.05). The last dosage (19.1 μg/μL) demonstrated a significant increase only in the event of late apoptosis (*p* < 0.01 vs. control). However, the second dosage, 10.7 μg/μL, registered a significant elevation of early (*p* < 0.001) and late (*p* < 0.01) apoptotic cells in comparison to untreated cells. Furthermore, compared to cells treated just with extract, a substantial rise in cells undergoing early apoptosis was seen in the groups treated with both extract and antibiotic, with the second dose showing the highest percentage. Additionally, this dosage recorded the highest percentage of early and late apoptosis among all tested groups. This finding may indicate that the extract lacks the capacity to counteract the gentamicin-induced programmed cell death that has been established. Therefore, more research is required to determine the exact mechanism of action of *Sorbus aucuparia* L. extract in relation to renal damage. Figure 4B,C provide a detailed reproduction of these results.

In terms of necrosis, *Sorbus aucuparia* L. extract determined a significant increase when compared to control group at all concentrations (*p* < 0.001). Gentamicin administration also induced a signifcant elevation of necrosis in comparison to untreated batch (*p* < 0.001). The co-treatment of gentamicin and *Sorbus aucuparia* L. extract determined a non-significant decrease in the number of necrotic cells at the lowest concentration (*p* > 0.05). Importantly, a considerable decline in the percentage of necrotic cells was noticed at the second dosage (*p* < 0.001), confirming once again the potential renal cell-protective effect of this extract at moderate doses. However, the 19.1 μg/μL concentration recorded a significant rise in the number of necrotic cells in contrast to gentamicin-only stressed cells (*p* < 0.001). Thus, we can deduce that modest doses of *Sorbus aucuparia* L. extract may reduce the number of necrotic cells in gentamicin-induced renal cell damage. More research is required in order to discover the precise toxic dose of the extract. Figure 4D offers a thorough replication of these findings.

## 3. Discussion

The current study aimed to investigate the antioxidant, antibacterial and cell-protective properties of bioactive compounds extracted from Romanian *Sorbus aucuparia* L. fruits. Consequently, in order to characterize the phytochemicals contained in these berries, both quantitative and qualitative measurements were conducted on fruit extracts. As a result, the quantitative determination revealed that the fruits contain phenolic compounds (1.11 ± 0.030 mg GAE/g DM), flavonoids (430.06 ± 2.603 µg QE/g DM) and carotenoids (95.68 ± 0.297 µg/g DM). In order to determine whether *Sorbus aucuparia* L. Romanian berries possess an adequate quantity of these constituents, our results were contrasted to other investigated varieties. As such, the total amount of phenolic compounds was comparable to that of Estonian, Russian, Turkish, Ukrainian and certain Polish cultivars [19,28,29,30,31,32]. However, the majority of Polish cultivars contain a higher level of polyphenols, together with several varieties from Serbia, Montenegro and Moldova [15,17,33,34,35]. Nevertheless, several Serbian types were deficient in these active chemicals compared to our study [36]. Moreover, the overall flavonoid content corresponded to that reported in most cultivars (Serbian, Montenegrin, Moldovan, Polish, Russian and Romanian) [15,17,29,37,38]. On the other hand, the total flavonoid concentration in the Ukrainian variants was lower than that in our investigation [31]. In terms of total carotenoids, the results were similar to those of Latvian cultivars [18]. Carotenoids were found in higher concentrations in the Moldovan variations as well as lower concentrations in the Serbian and Montenegrin types [17,35].

Furthermore, the qualitative assessment (HPLC-DAD-ESI-MS) was used to evaluate a more thorough characterization of the *Sorbus aucuparia* L. Romanian cultivars. Consequently, ten bioactive chemicals from three phenolic subclasses were identified. The most predominant phenols were quantified to be chlorogenic and neochlorogenic acid (704.79 µg/mL and 376.61 µg/mL, respectively). In accordance with earlier research, the main phenolic compounds discovered in *Sorbus aucuparia* L. fruits were represented by these acids [9,12,16,34,35]. Since both chemicals were identified in all analyzed *Sorbus aucuparia* L. cultivars, regardless of the geographic region, they were formerly suggested as indicators of the phytochemical and antioxidant profiles of *Sorbus* berries [9,39]. Moreover, other phenolic acids discovered in *Sorbus aucuparia* L. fruits were gallic and ferulic acids, at a concentration of 61.79 µg/mL and 45.01 µg/mL, respectively, being higher than those previously reported in some studies [9,17]. Among flavonoids, Q 3-O-rutinoside (rutin) was identified, having a concentration of 12.19 µg/mL. This amount is similar to that reported by Rutkowska et al. [34], while other authors registered higher quantities of this flavonol [9,16]. In addition, Q 3-O-glucoside (isoquercetin) was found in lower concentrations than previously reported [5,34]. Regarding the anthocyanin class, cyanidin 3-O-glucoside was discovered in a higher quantity than earlier published data [12,40]. However, this class was the least numerous in our study.

Thus, it can be concluded that the Romanian cultivars contain great amounts of bioactive chemicals, being consistent with the majority of previously published data. However, the cultivars’ chemical composition may vary depending on a multitude of factors, including genotypes, habitats, and the maturation stage.

Due to the fact that these fruits have confirmed their nutritional significance, through their abundance in bioactive chemicals, we opted to further explore their antioxidant potential. This was accomplished by conducting DPPH and FRAP assays and correlating our findings with those of other examined varieties. As a consequence, we measured an IC_50_ value of 24.51 ± 0.577 mg/mL. Several Serbian cultivars registered an IC_50_ of 0.08 ± 0.01 mg/mL [36], while Šavikin et al. [35] reported a higher antioxidant activity for Serbian and Montenegrin varieties (0.33 ± 0.04–4.26 ± 0.23 mg/mL). Olszewska et al. [32] registered a value of 6.44 ± 0.06 mg/mL for Polish cultivars. Additionally, a prior study from Romania showed IC_50_ to be 0.93 mg/mL [37]. Additionally, the IC_50_ ranged from 0.03 to 3.71 mg/mL for Canadian cultivars [41]. In essence, our result appears to be greater than previously published research. Regarding the FRAP assay, a value of 0.016 ± 1.047 µM TE/mL was obtained. Previous studies performed on Polish, Lithuanian and Turkish variations revealed FRAP values of 347 µM TE/g, 118 µM TE/mL and 29.3–56.8 µM TE/g, respectively [9,32,42]. Our outcome was significantly reduced in comparison to these published results. Thereby, we might assume that Romanian *Sorbus aucuparia* L. fruits shown a promising antioxidant activity. Additionally, considering all of the above listed factors, these differences may be related to the varying amounts of bioactive substances found in *Sorbus aucuparia* L. cultivars.

Natural products’ antimicrobial activity represents the topic of extended studies aiming to establish viable alternatives to classical antibiotics. For this reason, we decided to investigate this characteristic of *Sorbus aucuparia* L. fruits from Romania, which were found to contain noteworthy levels of biologically active chemicals with antioxidant function. As such, the findings pointed out in vitro bactericidal efficacy against all the bacterial strains, with a higher potential towards the Gram-positive: Methicillin-Susceptible *Staphylococcus aureus*, MRSA—Methicillin-Resistant *Staphylococcus aureus* and *Enterococcus faecalis*. Earlier studies contain data describing this property for extracts produced from different varieties of *Sorbus aucuparia* L. [5,43]. Such studies investigated extracts obtained from *Sorbus aucuparia* L. fruits [19,34], fruits and leaves [43], fruit pomace and juice [9,44]. A limited number of scientific works described the antibacterial efficacy of *Sorbus aucuparia* L. isolated fractions or compounds [45] and suggested polyphenols as the group responsible for the therapeutical efficacy [34,41]. The contradictory information refers to the spectrum of antimicrobial efficacy. While some authors reported in vitro efficacy against both Gram-positive and Gram-negative bacteria (*Pseudomonas aeruginosa* and *Citrobacter freundii*) [9], others present a rather modest or even weak effect against either the Gram-positive [34,41] or Gram-negative [5] *Staphylococcus aureus*. Additionally, the antimicrobial activity is described as bacteriostatic [41]. These differences can be explained by considering the methods and solvents employed to prepare the extracts, the chemical composition, and the concentration of the extracts. In this regard, an acetone extract was found with a better antimicrobial efficacy compared to the water and ethanol extracts [9]. In essence, the antimicrobial potential was previously reported, but the available literature could still include more comprehensive studies. Nonetheless, to our knowledge, this is the first study to evaluate the antibacterial property of the ethanolic extract obtained from *Sorbus aucuparia* L. fruits available in Romania.

Gentamicin is an antibacterial drug from the aminoglycoside class that is particularly effective against severe Gram-negative pathogens which have become resilient to other antibacterial agents. Despite gentamicin’s potent antibacterial properties, its use is limited mainly due to its propensity to induce nephrotoxicity as an adverse effect [46,47]. A major contributing factor to the nephrotoxicity induced by gentamicin is considered to be oxidative stress [48]. Hence, it has been established that a variety of antioxidants mitigate the deleterious consequences of oxidative damage. Numerous herbs exhibit antioxidant benefits since they contain great concentrations of bioactive components, including carotenoids and polyphenols [49]. Examples of these antioxidant-rich herbs that have recently proven to exert a protective impact in kidney injury brought on by gentamicin include *Cistanche deserticola* L., *Hedyotis aspera* L., and *Artemisia annua* L. [50,51,52]. As already stated, a plant with an increased quantity of bioactive constituents that possess antioxidant qualities is *Sorbus aucuparia* L. The components from *Sorbus aucuparia* L. berries might attenuate the gentamicin stress caused to renal tissue, a possible application of this plant for which little research has been conducted. Therefore, we thought it was essential to conduct a more in-depth analysis of these fruits’ cytoprotective impact on renal epithelium cells stressed with gentamicin. Cell viability tests, specifically MTT and Annexin V-FITC staining, were used to examine this effect.

Consequently, the MTT assay revealed a considerable reduction in cell viability in the gentamicin-treated group, indicating the effective establishment of an in vitro experimental model of nephrotoxicity. Following *Sorbus aucuparia* L. extract administration to gentamicin-stressed cells, a notable rise in cell viability was observed at the first two concentrations, displaying a protective impact against gentamicin’s oxidative effect. In contrast, the last concentration registered an even lower percentage of cell viability when compared to antibiotic-only treated cells. This finding could be a consequence of the complex interaction between carotenoids and reactive oxygen species. Additionally, the pro-oxidative impacts of high dosages of carotenoids have been observed in several in vitro and in vivo studies and are frequently linked to the build-up and ensuing detrimental effects of the resultant breakdown products. However, the properties of these generated products remain largely unknown [53]. Furthermore, we chose to compare our findings with those of other medicinal plants that may have a nephroprotective effect since, to the best of our knowledge, the effect of *Sorbus aucuparia* L. fruit extract on stressed renal epithelial cells in mice has never been previously investigated. Thereby, in a study by Guru et al. [54], gentamicin treatment resulted in an extensive reduction in cell viability (58%), whereas daidzein therapy exhibited a cytoprotective effect in a dose-dependent manner, with the highest concentration showing a cell viability of 90%. Additionally, similar results were obtained in the study conducted by Jasti et Bhikshapathi [55]. On the contrary, in our investigation, the lowest concentration provided the greatest viability, which declined as the extract’s concentration increased. Moreover, in another study, gentamicin significantly reduced cell viability by up to 25–50%. There was a noticeable improvement in the cytotoxicity following the administration of epigallocatechin gallate [56]. The results of our investigation are generally consistent with this previously published paper.

Apoptosis and necrosis are two of the cell death processes encountered during renal tubular damage [57]. The natural physiological reaction of cells to various events, infections, or damage—such as cytotoxic drug treatments or radiation therapy regimens—that result in irreversible DNA damage is called apoptosis [58]. It is an inactive mode of cell death that maintains the surrounding cellular environment intact and is crucial for an organism’s growth and homeostasis. The impacted cells exhibit condensing of chromatin, size reduction, and fragmentation into apoptotic bodies, unaccompanied by widespread inflammation [59,60]. Necrosis, on the other hand, is a disorderly and chaotic phenomenon that is thought to be an inadvertent way of cell destruction. Cell expansion, mitochondrial membrane permeability, and breakage of membranes that outcomes in the release of proinflammatory chemicals and cellular contents are its distinguishing characteristics [60,61]. As such, maintaining the plasma membrane intact, together with the quick dissolution of apoptotic bodies by local defense cells minimizes inflammatory cell enrollment, which would subsequently exacerbate tissue damage [61].

Related to these aspects, our results on flow-cytometry analysis of cell apoptotic rate showed a drastic decrease in cellular viability in the gentamicin-treated group. After *Sorbus aucuparia* L. extract treatment, there was a substantial increase in cell vitality at all concentrations, exhibiting a cytoprotective effect comparable to that of the group that received extract therapy alone. The last concentration, however, registered the greatest percentage of necrotic cells, which was more prominent than in the gentamicin-stressed cells. This was most likely due to the extract’s high dosages and gentamicin’s additional pro-oxidant actions. These findings are largely consistent with those described in the MTT assay. Furthermore, because this is the first research to our information that analyzes the influence of *Sorbus aucuparia* L. extract on cell apoptotic rate in an in vitro nephrotoxicity trial, we contrasted our results to those of other herbal remedies with potential kidney-protective properties. Thus, in a previously mentioned study, the flow cytometry analysis indicated that gentamicin increased the number of apoptotic cells, whereas using epigallocatechin gallate as a form of therapy avoided this aspect. [56]. In another study, gentamicin determined a significant increase in early and late apoptosis as well as necrosis of the kidney cells, whereas the concurrent treatment with gentamicin and ellagic acid resulted in a considerable reduction in apoptotic numbers of cells [62]. Our results are essentially similar to those of these earlier publications, except for the last concentration, which showed a considerably higher level of necrosis than the gentamicin-treated group. Moreover, a previous study conducted in our laboratory revealed that the gentamicin group registered drastic declines in cell viability and also the greatest rises of necrosis. Following the co-treatment of gentamicin and *Cornus mas* L. extract, a substantial diminution of cell apoptosis was observed at the first two concentrations. However, the largest dosage was shown to be highly toxic to cells, indicating a lower viability rate than cells that were exposed to the antibiotic alone [63]. The current study’s findings are mostly in line with the outcomes of this previous inquiry. However, the highest concentration of *Sorbus aucuparia* L. extract determined a more pronounced cytotoxic impact compared to *Cornus mas* L. extract, due to the presence of a higher percentage of necrotic cells.

In a nutshell, it should be highlighted that this is the first publication to demonstrate that *Sorbus aucuparia* L. Romanian cultivars protect primary renal cell lines during gentamicin stress. This research may spark further consideration in minimizing renal damage induced by synthetic antibiotics.

## 4. Materials and Methods

### 4.1. Chemical Agents, Bacterial Strains and Cell Culture

Phenolic and carotenoid components’ standards were supplied by Sigma-Aldrich (Darmstadt, Germany). Plant Flavonoids Colorimetric Assay Kit was obtained from Elabscience Biotechnology Inc. (Houston, TX, USA). Prior to HPLC screening, each of the analyzed samples underwent filtration using a 0.45 µm MF-Millipore™ Membrane Filter acquired from Merck (Darmstadt, Germany). The bacterial reference strains: *Staphylococcus aureus* ATCC 25923, *Staphylococcus aureus* ATCC 700699, *Bacillus cereus* ATCC 14579, *Enterococcus faecalis* ATCC 29219, *Escherichia coli* ATCC 25922 and *Pseudomonas aeruginosa* ATCC 27853 were purchased from Oxoid Ltd. (Hampshire, UK). Culture mediums for bacteria were purchased from Merck (Darmstadt, Germany). Sigma-Aldrich (St. Louis, MO, USA) provided the enzymatic mixtures for cell cultures, while Gibco Life Technologies (Paisley, UK) provided the culture medium constituents. Thermo Fisher Scientific Inc. (Waltham, MA, USA) provided the Annexin V-FITC with propidium iodide (PI) flow-cytometry Kit.

### 4.2. Fruit Collection and Extraction

**Figure 5:**
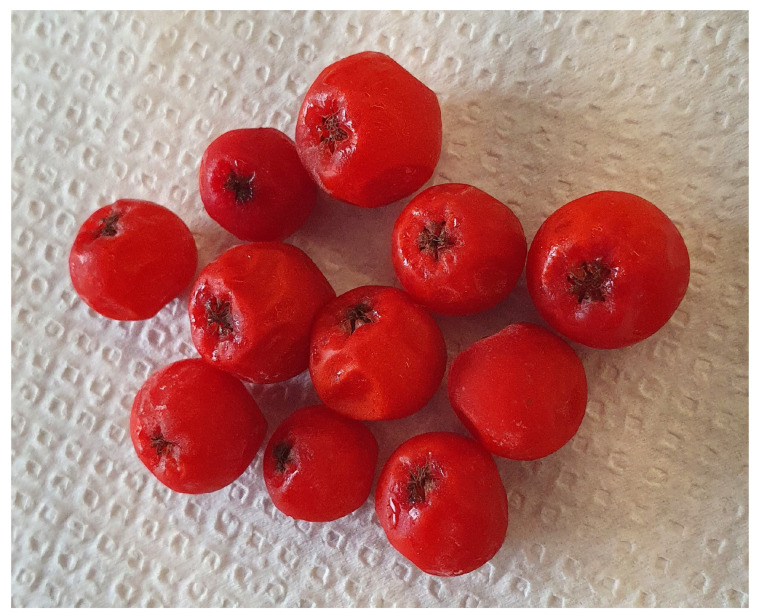
*Sorbus aucuparia* L. wild fruits.

Between August and September of 2020, *Sorbus aucuparia* L. branches with leaves and fruits were manually harvested from shrubs growing in Mărişel commune’s high peaks (46°40′03.7″ N 23°06′35.6″ E, Cluj County, Romania) (Figure 5). Following plant recognition, the limbs, leaves and stones were meticulously separated, and the entire amount of fruit flesh was equally distributed in 10 g sampling bags and frozen at −18 °C until subsequent analysis.

A TEESA TSA3031 dryer (Lechpol Electronics Leszek Sp. K., Garwolin, Poland) adjusted to 45 °C had been employed to defrost and dry the berries for one week, with their subsequent mincing into a powdered material. Following fruit pulverization, an ethanolic extract was prepared in accordance with earlier research performed in our lab [63]. Briefly, a mixture of 10 g powder and 100 mL ethanol 96% was homogenized (2 homogenizing steps of 2 h at 1000 rpm using a magnetic stirrer–VELP Scientifica, Monza-Brianza, Italy), filtered and concentrated by evaporation, using a 45 °C Eppendorf Concentrator Plus evaporator (Eppendorf, Hamburg, Germany). Finally, the acquired extract volume was quantified. All extraction steps were carried out in three replicates.

Furthermore, at the suggestion of the reviewed literature that these fruits are abundant in lipophilic substances [5,17], specifically carotenoids, which are significantly less soluble in alcohol, we considered it would be interesting to prepare a specific extraction of carotenoids. As such, 5 g of fruit powder was extracted using a 90 mL mixture of methanol, ethyl acetate and petroleum ether (1:1:1). Subsequent to homogenization, filtration, and re-extraction, the filtrates were collected in a separatory tube and progressively partitioned using water, diethyl ether and saturated saline solution. Following residual water removal, the mix was subjected to rotating evaporation at 40 °C. Lastly, the resulting concentrated oleoresin was reconstituted with a specified volume of dimethyl sulfoxide (DMSO) in order to obtain the final extract. The extraction method was performed in triplicate.

Analyzes performed on the ethanolic extract were represented by the quantitative and qualitative determination of phenolic compounds by spectrophotometric and chromatographic methods, and the in vitro evaluation of the antibacterial effect. The carotenoid extract was utilized to determine total carotenoids and assess the antioxidant activity in vitro. Additionally, due to the presence of considerable amounts of carotenoids, which contribute significantly to the fruits’ antioxidant property, we opted to employ this extract in the in vitro study on renal cell cultures.

### 4.3. Determination of Total Polyphenols, Flavonoids and Carotenoids

These experiments were carried out according to the previously mentioned study [63]. In brief, the total amount of phenolic compounds was assessed using the Folin–Ciocalteu technique and measured at 765 nm. After obtaining a gallic acid calibration curve, with concentrations spanning from 50 to 450 µg/mL, the results were quantified and given in mg of gallic acid equivalents (GAE) per mL of extract and per gram of dry mass (g DM).

Furthermore, a colorimetric assay kit (Plant Flavonoids Colorimetric Assay Kit—Elabscience Biotechnology Inc., Houston, TX, USA) was used to determine the total flavonoid concentration. The prepared samples were measured at 510 nm and further calculated using a quercetin calibration curve, containing six levels of concentration in the range of 20–150 µg/mL. The outcomes were presented in µg of quercetin equivalents (QE) per mL of extract and per gram of dry mass (g DM).

The total carotenoid content was determined using an UV–Vis spectrophotometric assay. The absorbance spectrum of the extract was detected in the range of 350–700 nm. The samples’ absorbance was read at 467 nm. The results were calculated based on a mathematical formula: x(g) = E × V/E^1%^_1cm_ × 1 × 100, where E = the wavelength of absorption, V = the final extract volume, E^1%^_1cm_ = the percentage extinction coefficient and 1 = the cuvette’s depth. Following quantification, the findings were revealed as µg per gram of dry mass (g DM).

Absorbance measurements were performed using a microplate spectrophotometer (SPECTROstar^®^ Nano—BMG Labtech, Ortenberg, Baden-Württemberg, Germany). All assessments were performed in three replicates.

### 4.4. Identification of Phenolic Compounds by HPLC-DAD-ESI-MS Analysis

An Agilent 1200 HPLC system (model 1160–Agilent Technologies, Carpinteria, CA, USA) provided with a photodiode UV–Vis detector (DAD) linked to a single mass detector (MS) was utilized to chromatographically isolate the phenolic constituents. Following the procedures described by Dumitraş et al. [64], the protocol for compound separation was applied. Standard curves of HPLC-grade reagents such as chlorogenic acid, rutin and cyanidin were used. Phenolic acids were detected at 280 nm, flavonoids at 340 nm, and anthocyanins at 520 nm, in the 200–600 nm range of the spectrum data. Data were collected and analyzed using Agilent ChemStation software (Chelmsford, MA, USA) [63]. The measurements were performed in triplicate.

### 4.5. In Vitro Antioxidant Activity

The evaluation of the antioxidant capacity of the *Sorbus aucuparia* L. carotenoid extract was carried out using Radical Scavenging Activity (DPPH) and Ferric Reducing Antioxidant Power (FRAP) assays. These analyzes were performed in triplicate, according to an earlier study [63]. In brief, for the DPPH test, various concentrations of 2 mL extract were combined with 2 mL DPPH concentrated solution (0.10 g/L). Following sample preparation, the absorbance change was measured at 517 nm. Results were calculated based on a mathematical formula: (A blank—A tested extract/A blank) × 100 = DPPH inhibition %, where A = absorbance, and were further expressed as IC_50_ mg/mL.

Regarding the second method, FRAP reagent was formulated utilizing established volumes of TPTZ, ferric chloride, and acetate buffer solutions. Consequently, in 4 mL of extract, 6 mL of FRAP reagent were combined with 0.8 mL of purified water. Absorbance measurements were performed at 593 nm. A Trolox calibration curve was used for calculation. The results were displayed in µmol Trolox equivalents (TE)/mL of extract.

### 4.6. In Vitro Antibacterial Capacity

The *Sorbus aucuparia* L. ethanolic extract was tested for its in vitro antibacterial properties employing an agar-well diffusion method [65] based on EUCAST (European Committee on Antimicrobial Susceptibility Testing) criteria [66]. The set of reference bacteria included: *Staphylococcus aureus* ATCC 25923 (methicillin-susceptible *S. aureus*, MSSA), *Staphylococcus aureus* ATCC 700699 (methicillin-resistant *S. aureus*, MRSA), *Bacillus cereus* ATCC 14579, *Enterococcus faecalis* ATCC 29219, *Escherichia coli* ATCC 25922 and *Pseudomonas aeruginosa* ATCC 27853. The protocol was performed in accordance with Niculae et al. [67]. For evaluation, negative (96% ethanol in water *v*/*v*) and positive controls (standard antibiotics disks: amoxicillin-clavulanic acid (20–10 µg), gentamicin (10 µg) (Oxoid Ltd., Hampshire, UK) were tested. After 24 h of incubation at 37 °C, the agar plates containing analyzed samples were checked to record the values of the diameter inhibition zone (in mm).

The *Sorbus aucuparia* L. extract was further investigated using the broth microdilution method [68]. Based on this assay, values for the minimum inhibitory (MIC) and bactericidal (MBC) concentrations were determined. The procedures were implemented in compliance with the previous research [63]. For interpretation, the turbidity absence in the well was indicative of the inhibitory effect, and the lowest concentration was recorded as the MIC value. Additionally, the absence of bacterial growth (no colonies) was indicative of the bactericidal effect, and the lowest concentration was recorded as the MBC value. Once the MIC and MBC were determined, their values were used to calculate the MIC index as the ratio MBC/MIC. According to this parameter, the extract’s antimicrobial efficacy can be bactericidal (MBC/MIC ≤ 4) or bacteriostatic (MBC/MIC > 4) [65]. Gentamicin 50 mg/mL (Sigma-Aldrich, St. Louis, MO, USA) and MH broth were tested as controls.

### 4.7. Experimental Procedures, Laboratory Rodents and Primary Renal Epithelial Cell Cultures

The research was undertaken in the authorized Biobase of the Faculty of Veterinary Medicine, Cluj-Napoca, Romania. The ethical board of the University of Agricultural Sciences and Veterinary Medicine, Cluj-Napoca, Romania (no. 256/21.04.2021) and the Regional Sanitary Veterinary and Food Safety Authority (no. 274/12.11.2021) validated this study. Additionally, all animal practices followed Directive 63/2010/EU and legislative Act no. 43/2014. As such, one mature, five-days pregnant female C57BL/6J mouse, holding 27 g, was purchased from the Oncological Institute “Prof. Dr. Ion Chiricuţă”, Cluj-Napoca, Romania, and employed for the study. The individual had been acclimating in the research facility for seven days prior to the study’s procedures. Importantly, the housing standards were designed in compliance with Directive 63/2010/EU and ISO 10993-6 criteria [69]. At the conclusion of the acclimatization stage, which coincided with the 13th day of pregnancy, the female was generally anesthetized and compassionately sacrificed through dislocation of the cervical vertebrae. Following euthanasia, the abdominal cavity and the uterus were incised, with the removal of 6 fetuses that were isolated and cleaned in Dulbecco’s phosphate-buffered saline (PBS). The abdomens of the fetuses were also surgically sliced, and the kidneys were removed and placed in PBS solution for subsequent use. The surgical procedures adhered to ISO 10993-6 specifications [63,69].

To grow primary renal cultures, cells were extracted from the kidneys of mouse fetuses using a combined technique that included tissue explants and enzymatic treatment, being described in detail in a previous paper [63]. The subculturing process was concluded at 5 × 10^3^ cells/plate.

### 4.8. In Vitro Cell Viability Assay

To evaluate the extract’s potential cell-protective effect, the MTT (3-(4,5-dimethylthiazol-2-yl)-2,5-diphenyltetrazolium bromide) colorimetric assay was employed. The protocol was carried out in accordance with previously mentioned research [64]. Three different concentrations of the extract to be tested—SA1, SA2, and SA3—in quantities of 10, 15, and 20 μL were used. These volumes were calculated using the total carotenoid content present in the analyzed extract. Therefore, SA1 contains 4.8 µg/µL carotenoids, SA2 contains 10.7 µg/µL carotenoids, and SA3 contains 19.1 µg/µL carotenoids. Additionally, some cell cultures underwent treatment with a 100 mg/mL gentamicin solution in 3 distinct dosages, namely GEN1 = 100 μg/μL, GEN2 = 150 μg/μL, and GEN3 = 200 μg/μL; other cultures received a combination treatment of *Sorbus aucuparia* L. extract and gentamicin (GSA1 = SA1 + GEN1, GSA2 = SA2 + GEN1, and GSA3 = SA3 + GEN1). Cells left untreated served as the negative control, whereas cells exposed to gentamicin served as the positive control. Following sample preparation, the chromogenic reaction’s intensity was recorded at 450 nm using the BioTek Synergy 2 spectrophotometer (Winooski, VT, USA). Finally, an equation was used to compute the percent of cellular viability: A_treated-cells_/A_control cells_ × 100 = viable cells%; A = absorbance [63]. Three separate runs of these assessments were conducted.

### 4.9. In Vitro Cell Apoptosis Assay

Cell death score was assessed utilizing the Annexin V-FITC cell apoptosis detection kit (Thermo Fisher Scientific Inc., Waltham, MA, USA). The analysis was made in accordance with the guidance included in the package and employed the identical substances and concentrations mentioned in the MTT experiment. After applying the assay protocol, the samples were subjected to FACS analysis (fluorescence sorting of activated cells), using the same flow-cytometer as mentioned in the prior study [63]. The FACSDiva 6.1.2 program (Becton Dickinson, San Jose, CA, USA) was implemented to analyze the dataset. The interpretation was performed as follows: Annexin V-FITC+ and PI− represent early apoptosis, V-FITC+ and PI+ illustrate late apoptosis, V-FITC− and PI+ indicate necrosis and Annexin V-FITC− and PI− represent viability [63,64]. The measurements were carried out in triplicate.

### 4.10. Statistical Analysis

Following each examination in triplicate, the outcomes were presented as mean ± standard deviation. The analysis of the data was accomplished by employing a statistical application, namely Graph Pad Prism 8 (San Diego, CA, USA). One-way ANOVA was used to compute and interpret the results. The significance threshold was set at *p* < 0.05.

## 5. Conclusions

This paper represents an extensive analysis of the phytochemical profile, antioxidant and antibacterial effects of *Sorbus aucuparia* L. fruit extracts; it is also, to the best of our knowledge, the first article outlining its cytoprotective properties on stressed mice renal cells in vitro. The fruits demonstrated to be abundant in bioactive compounds, specifically phenolic compounds and carotenoids. Using HPLC analysis, ten compounds of three phenolic subclasses were identified, from which chlorogenic and neochlorogenic acids were found in the largest amounts. Additionally, *Sorbus aucuparia* L. fruit extracts exhibited a noteworthy in vitro antioxidant capacity and antibacterial activity towards all analyzed microbial strains. Furthermore, except for the last concentration, which displayed pro-oxidant effects similar to that of gentamicin, it also showed cell-protective impacts on primary mouse kidney cells. Without diminishing the importance of this paper, more research is required to clarify the underlying mechanisms of cytoprotection and determine the harmful extract dosage. Nevertheless, these findings might create new scientific challenges regarding the development of nutraceuticals that attenuate drug-induced kidney disease.

## Figures and Tables

**Table 1:** HPLC-DAD-ESI-MS identification of phenolic compounds of *Sorbus aucuparia* L. fruit ethanolic extract.

Peak No.	Retention Time R_t_ (min)	UV λ_max_ (nm)	[M+H]^+^ (*m*/*z*)	Compound	Subclass	Concentration (µg/mL)
1	3.31	270	**333**, *171*	Gallic acid-glucoside	Hydroxybenzoic acid	61.799
2	3.89	520, 322, 280	**611**, *449*	Cy 3-O-(caffeoyl-glucoside)	Anthocyanin	3.212
3	9.74	330	**355**, *163*	4-Caffeoylquinic acid (Cryptochlorogenic acid)	Hydroxycinnamic acid	91.199
4	10.39	330	**355**, *163*	3-Caffeoylquinic acid (Neochlorogenic acid)	Hydroxycinnamic acid	376.610
5	11.82	520, 280	**449**, *287*	Cy 3-O-glucoside	Anthocyanin	19.237
6	12.16	330	**355**, *163*	5-Caffeoylquinic acid (Chlorogenic acid)	Hydroxycinnamic acid	704.792
7	14.45	360, 255	**627**, *303*	Q 3,4′-O -diglucoside	Flavonol	70.310
8	15.76	360, 255	**611**, *303*	Q 3-O-rutinoside (Rutin)	Flavonol	12.198
9	16.32	361, 251	**465**, *303*	Q 3-O-glucoside	Flavonol	24.139
10	17.26	322	**195**	Ferulic acid	Hydroxycinnamic acid	45.018

The mean of three replicate results was provided in µg/mL of extract; Cy = Cyanidin, Q = quercetin.

**Table 2:** In vitro antibacterial activity of *Sorbus aucuparia* L. ethanolic extract using the well diffusion method.

Tested Products	Diameters of Inhibition Zone (mm)					
	MSSA	MRSA	*Bacillus* *cereus*	*Enterococcus faecalis*	*Escherichia coli*	*Pseudomonas* *aeruginosa*
*Sorbus aucuparia* L.	27.33 ± 0.47 ^a,d^	24.00 ± 0.00 ^a,c^	18 ± 0.00 ^a,d^	25.67 ± 0.94 ^a,c^	25.67 ± 0.47 ^a,c^	17.67 ± 0.47 ^b,c^
Gentamicin	20 ± 0.00	17 ± 0.00	26 ± 0.00	0	19 ± 0.00	19 ± 0.00
Amoxicillin- clavulanic acid	29 ± 0.00	28 ± 0.00	20 ± 0.00	17 ± 0.00	19 ± 0.00	0

MSSA—Methicillin-Susceptible *Staphylococcus aureus*, MRSA—Methicillin-Resistant *Staphylococcus aureus*. Values are presented as means of triplicate determinations (n = 3) ± standard deviations. Lowercase letters in the same column indicate significant differences: ^a^ *p* < 0.05 (extract vs. Gentamicin); ^b^ *p* > 0.05 (extract vs. Gentamicin); ^c^ *p* < 0.05 (extract vs. Amoxicillin-clavulanic acid), ^d^ *p* > 0.05 (extract vs. Amoxicillin-clavulanic acid); Gentamicin (10 μg/disk) and Amoxicillin-clavulanic acid (25–10 μg/disk) were used as positive controls (antibiotics).

**Table 3:** In vitro antibacterial activity of *Sorbus aucuparia* L. ethanolic extract using broth microdilution assay.

Tested Products	MIC Index MBC/MIC					
	MSSA	MRSA	*Bacillus* *cereus*	*Enterococcus* *faecalis*	*Escherichia* *coli*	*Pseudomonas* *aeruginosa*
*Sorbus aucuparia* L.	1 D7/D7	1 D7/D7	1 D6/D6	1 D7/D7	1 D5/D5	1 D4/D4

D4—7 represent the serial dilutions of *Sorbus aucuparia* L. ethanolic extract which are related to the total polyphenol concentration; D4—0.087 µg GAE/uL, D5—0.043 µg GAE/uL, D6—0.021 µg GAE/uL, D7—0.010 µg GAE/uL. The MIC index value indicates in vitro bactericidal (MBC/MIC ≤ 4) or bacteriostatic (MBC/MIC > 4) efficacy.

## Data Availability

The data presented in the study are available in the article.
